# Controversies in ACL revision surgery: Italian expert group consensus and state of the art

**DOI:** 10.1186/s10195-022-00652-9

**Published:** 2022-07-15

**Authors:** Fabrizio Matassi, Niccolò Giabbani, Enrico Arnaldi, Alessandro Tripodo, Giovanni Bonaspetti, Corrado Bait, Mario Ronga, Paolo Di Benedetto, Stefano Zaffagnini, Eugenio Jannelli, Alfredo Schiavone Panni, Massimo Berruto

**Affiliations:** 1 Orthopaedic Clinic CTO, University of Florence, Florence, Italy; 2 Center of Knee Surgery, Humanitas, Milan, Italy; 3 San Camillo Institute, Forte dei Marmi, Lucca, Italy; 4 U.O. Ortopedia e Traumatologia 2, Istituto Clinico Sant’Anna, Brescia, Italy; 5 Villa Aprica Institute, Como, Italy; 6 Orthopaedic and Trauma Operative Unit, Department of BIOMORF, University Hospital G. Martino, University of Messina, Messina, Italy; 7 Orthopaedic Clinic, University Hospital of Udine, Udine, Italy; 8 IRCCS Istituto Ortopedico Rizzoli, Bologna, Italy; 9 Department of Medical and Surgical Specialties and Dentistry, University of Campania “Luigi Vanvitelli”, Naples, Italy; 10 UOS Knee SURGERY-1st University Clinic of Orthopaedics, ASST Pini-CTO, Milan, Italy

**Keywords:** Anterior cruciate ligament revision, Anterior cruciate ligament reconstruction, ACLR failure, Revision, Survey, Consensus, Italian expert group

## Abstract

**Background:**

Revision ACL reconstruction is a complex topic with many controversies and not-easy-to-make decisions. The authors’ aim is to provide some feasible advice that can be applied in daily clinical practice with the goal of facilitating the decision-making process and improving the outcomes of patients subjected to revision ACL reconstruction.

**Methods:**

A national survey with seven questions about the most controversial topics in revision ACL reconstruction was emailed to members of two societies: SIOT and SIAGASCOT. The participants’ answers were collected, the most recent literature was analyzed, and a consensus was created by the authors, according to their long-term surgical experience.

**Conclusions:**

The decision-making process in revision ACL reconstruction starts with a standardized imaging protocol (weight-bearing radiographs, CT scan, and MRI). One-stage surgery is indicated in almost all cases (exceptions are severe tunnel enlargement and infection), while the choice of graft depends on the previously used graft and the dimensions of the tunnels, with better clinical outcomes obtained for autografts. Additional procedures such as lateral extra-articular tenodesis in high-grade pivot-shift knees, biplanar HTO in the case of severe coronal malalignment, and meniscal suture improve the clinical outcome and should be considered case by case.

**Level of evidence:**

V (Expert opinion).

## Introduction

Revision ACL reconstruction (revACLr) is becoming an increasingly common procedure as the number of primary anterior cruciate ligament reconstructions (ACLr) continues to rise [1, 2]. Large multicenter cohort studies have described revision rates of ACLr of 1.7–7.7%, with a 5-year survival rate of around 95.4%. Among patients undergoing revACLr, the clinical outcomes are inferior compared to primary ACLR and the re-revision rate ranges from 2.0 to 8.9% [3–6].

Many studies have identified some risk factors for revACLr, such as male sex, young age, and elevated body mass index as well as an early return to sport and pivoting sport after the reconstruction. However, traumatic re-injury is only the tip of the iceberg; there are also many technical factors, such as tunnel malpositioning, inadequate graft fixation, graft choice for primary reconstruction, insufficient anterolateral structures, and an elevated/excessive posterior tibial slope [7–10].

In clinical practice, there are a lot of controversies and a lack of univocal topics regarding revACLr surgery, and there are numerous potential pitfalls during surgery. The diagnostic exams to be made, the most appropriate preoperative planning, when to perform a one-stage or a two-stage procedure, the choice of the graft, and the need for lateral augmentation are just some of the questions that orthopedic surgeons have to face during their practice.

There is no univocal evidence available in the literature regarding these main topics in revACLr, so it is very often personal experience and the habits of the specific Institution that guide the decision-making process.

Although best clinical practice guidelines are a fundamental tool for health-care providers, the paucity of the literature on the complex field of revACL surgery does not allow strong evidence-based recommendations to be made.

The purpose of the present study is to focus on the main controversial topics in ACL revision surgery. We collected data on the surgical experiences of knee-specialized Italian orthopedic surgeons (expert group) and high-volume knee surgeons who are members of SIOT (the Italian Society of Orthopaedics and Traumatology) and SIAGASCOT (the Italian Society of Arthroscopy, Knee, Upper Limb, Sports, Cartilage and Orthopaedic Technologies), who participated in a national survey. These data were analyzed taking into account the most recent and significant studies, and a consensus was created, leading to some feasible advice that can be used in daily clinical practice.

## Materials and methods

This study was elaborated by the authors through a national survey to investigate the Italian experience and perspective about surgical trends and the most ambiguous topics in revACL surgery. A national survey was emailed to all active and associate members of SIOT and SIAGASCOT from August 2021 to October 2021. The survey was completed by 51 surgeons and the data were registered and stored in a database. Demographic data on the participants (years of working experience, main field of interest, and personal experience of revACL surgery) are reported in Table 1.

**Table 1:** Demographic data of the participants

Participants (total: 51)	
How many years of working experience do you have?	
Resident	6 (11.8%)
0–5 years	8 (15.7%)
5–10 years	7 (13.7%)
> 10 years	30 (58.8%)
What’s your main field of interest?	
Knee prosthetic surgery and sport medicine	36 (70.6%)
Sport medicine	7 (13.7%)
Prosthetic surgery	5 (9.8%)
General traumatology	3 (5.9%)
How many revACLr surgeries per year do you perform?	
More than 10	20 (39.2%)
5–10	15 (29.4%)
2–5	8 (15.7%)
Less than 2	8 (15.7%)

The survey consisted of a questionnaire with seven questions, each with various possible answers. The questions dealt with the most controversial topics in ACL revision surgery, which were chosen because there was no univocal evidence on them in daily clinical practice and in the literature. The questions are listed in Table 2. The answers are summarized in Table 3.

**Table 2:** List of the questions

1	How many ACL revision surgeries do you perform with the one-stage technique and how many with the two-stage technique?
2	What diagnostic tests/tools do you use to plan an ACL revision surgery?
3	If a previous ACL reconstruction with patellar tendon fails, what’s your graft of choice? And if a previous ACL reconstruction with hamstring fails, what’s your graft of choice?
4	What is your technique to make the femoral tunnel?
5	What is the role of lateral extra-articular tenodesis (LET) in ACL revision surgery?
6	What do you do in the case of a patient with primary or secondary varus (according to the Noyes classification) with pain and instability secondary to failure of a previous ACL reconstruction surgery?
7	Which meniscal injuries do you treat in ACL revision surgery?

**Table 3:** Participants’ answers to the questions

Question		Answers			
1	How many revACLr surgeries do you perform with the one-stage technique and how many with the two-stage technique?	100% one-stage; 0% two-stage	90% one-stage; 10% two-stage	80% one-stage; 20% two-stage	70% one-stage; 30% two-stage
		24 (47.1%)	16 (31.4%)	10 (19.6%)	1 (1.9%)
2	What diagnostic tests/tools do you use to plan an ACL revision surgery?	AP and LL X-ray + WB X-ray	AP and LL X-ray + WB X-ray + stress X-ray	AP and LL X-ray + WB X-ray + CT	AP and LL X-ray + WB X-ray + CT + MRI
		6 (11.8%)	0 (0%)	11 (21.6%)	34 (66.7%)
3	If a previous ACL reconstruction with patellar tendon fails, what’s your graft of choice?	Hamstrings	Quadriceps tendon	Allograft	Synthetic graft
		33 (64.7%)	2 (3.9%)	16 (31.4%)	0 (0%)
	And if a previous ACL reconstruction with hamstring fails, what’s your graft of choice?	Patellar tendon	Contralateral hamstrings	Allograft	Synthetic graft
		29 (56.9%)	6 (11.8%)	16 (31.4%)	0 (0%)
4	What is your technique to make the femoral tunnel?	Trans-tibial	Transportal (antero-medial)	Outside-in	Decision case by case
		5 (9.8%)	10 (19.6%)	15 (29.4%)	21 (41.1%)
5	What is the role of lateral extra-articular tenodesis (LET) in ACL revision surgery?	I always perform	I always perform except in revACLr with patellar tendon	I perform only in cases with a high-grade pivot shift test	I perform only in young patients with a risk of re-injury
		19 (37.3%)	0 (0%)	29 (56.9%)	3 (5.9%)
6	What do you do in the case of a patient with primary or secondary varus (according to the Noyes classification) with pain and instability secondary to failure of a previous ACL reconstruction surgery?	revALCr + tibial osteotomy	Isolated revACLr	Isolated tibial osteotomy	First step: tibial osteotomy; second step: revACLr
		28 (54.9%)	5 (9.8%)	1 (2%)	17 (33.3%)
7	What meniscal injuries do you treat in ACL revision surgery?	Root lesion	Ramp lesion	Bucket handle lesion	Every type of lesion
		0 (0%)	0 (0%)	1 (2%)	50 (98%)

*AP* anteroposterior; *LL* laterolateral; *revACLr* anterior cruciate ligament revision

The second part of this study consisted of a systematic review of the recent literature (starting from 2016) and was performed using the PubMed and Cochrane Library electronic databases in accordance with the Preferred Reporting Items for Systematic Reviews and Meta-Analysis (PRISMA) guidelines [11]. The search terms were mapped to Medical Subject Headings (MeSH) terms where possible. Search terms were: ACL revision OR ACL failure OR ACL one-stage technique OR two-stage technique OR ACL planning OR femoral tunnel OR lateral extra-articular tenodesis OR graft type OR osteotomy OR meniscal lesion. Each term was then combined with the AND operator to produce the search strategy.

The surgeons’ answers and literature were therefore analyzed by the authors to create a consensus leading to practical advice regarding ACL revision surgery.

## Discussion

The following consensus represents a summary of the personal experience of a group of knee-specialized Italian orthopedic surgeons who matched with an analysis of the most recent literature regarding “hot topics” in revACLr. More than 68% of the surgeons who completed the survey were considered high-volume surgeons with a high level of experience (performing more than five revACLr surgeries per year), and thus the result of this survey represents a broad and reliable point of view regarding this complex topic.

One of the main factors that influence morbidity risk after revACLr is the surgeon’s annual volume of revACLr surgeries. Four cases per year is considered the threshold. Leroux et al., analyzing 827 cases of ACL revision, reported that high-volume surgeons yield improved outcomes, such as lower infection rates, transfusion rates, procedure times, and shorter lengths of patient hospital stay, compared to low-volume surgeons [5]. Sutherland et al., analyzing the risk factors for revACLr and the frequency with which patients change surgeons, found that patients are more likely to change surgeons if their primary ACLR was performed by a lower-volume surgeon [12].

RevACLr is a highly demanding procedure that must be addressed by experienced surgeons in order to standardize the operative and perioperative path and give the patients the best outcomes with the lowest risk of complications.

### How many ACL revision surgeries do you perform with the one-stage technique and how many with the two-stage technique?

From our analysis, most of the surgeons prefer one-stage revACLr, with 47.1% of the surgeons using the one-stage technique in all of their ACL revision cases, and 31.4% performing one-stage revACLr in 90% of the cases and two-stage revACLr in 10% of the cases.

The two-stage procedure consists of initial bone grafting of the tunnel to deal with malposition and achieve widening, followed by definitive ACL reconstruction after a few months. However, some studies reported that two-stage surgery is usually associated with a greater risk of cartilage damage and meniscal injury due to the long period of time for which the knee is left unstable and the longer return-to-play times in athletes. To avoid the drawbacks of a delayed reconstruction, there is interest in one-stage revision reconstruction, with recent studies showing significant improvements in patient function and comparable results in terms of graft failure and patient-reported outcomes [2].

White et al. reported a case series of 91 patients who underwent one-stage ACL revision surgery, where a decision-making algorithm was used to guide the choice of graft, fixation method, and surgical technique. They found that one-stage revision can be performed reliably in the majority of patients, with good clinical outcomes, low re-rupture rates, and high return-to play rates, even in the elite athlete population [2].

Another classification system that evaluates tunnel malposition and bone loss is described by Sa et al. It is based on CT scan images, and those authors proposed some technical solutions to deal with revACLr and help with the decision about whether to undertake one-stage or two-stage revision [13]. In particular, the two-stage procedure is indicated mainly in the case of tunnel enlargement greater than 16 mm or in the case of enlargement that is greater than 100% of the initial tunnel diameter. Furthermore, the two-stage technique is specifically indicated for an infection where the graft should be removed and the tunnel filled with graft, and in the case of a severe loss of range of motion [8].

Several graft options exist for bone tunnel augmentation in two-stage revACLr. From the literature analysis, 4 studies reported the use of an autograft (iliac crest bone autograft, *n* = 3 studies; tibial bone autograft, *n* = 1 study); 2 studies reported the use of an allograft (allograft bone matrix and allograft bone chips); and 1 study reported the use of a synthetic bone substitute [9]. In their systematic review regarding bone graft options in two-stage revACLr, they conclude that autologous bone is associated with a lower risk of ligament graft failure compared with allograft bone. An accurate method of ensuring adequate bone graft incorporation before the second stage of surgery needs to be clearly established, with some studies reporting the use of CT scan and others reporting the use of X-rays. The time interval between the first- and second-staged procedures was described as ranging from 3 to 8 months in 4 studies. The rehabilitation protocol between stages needs to be clearly established [9].

#### Summary

The majority of revACLr surgeries can be performed as a one-stage surgery after a thorough evaluation of the tunnel placement and widening (an algorithm is needed for decision making); two-stage revision is indicated for a large bone defect or tunnel collision, infection, and a loss of range of motion. For two-stage revision, there is poor evidence regarding the use of autograft bone to fill the bone tunnel. No agreement between studies is reported about the timing between the two stages, the method used to evaluate bone incorporation, and the rehabilitation protocol.

The cause of ACL failure should be taken into account, as it could provide guidance regarding the correct treatment: in the case of significant trauma on a correctly placed graft, we recommend one-stage revision (if feasible); when there is not significant trauma in malpositioned tunnels, two-stage surgery is usually (but not always) more appropriate.

However, the final decision on one-stage vs. two-stage surgery should be based on the actual intraoperative conditions of the tunnels; surgeons (and patients) must be aware of this.

### What diagnostic tests/tools do you use to plan a revACLr?

More than two-thirds of surgeons (66.7%) deem it necessary to perform preoperative weight-bearing radiographs, computed tomography (CT scan), and magnetic resonance imaging (MRI). Each of the following preoperative exams is necessary to analyze some different aspects when planning a revACLr:

- AP and lateral (full extension) radiographs of the knee and Rosenberg and axial views of the patella are useful for evaluating tunnel position, tibial slope, and presence of arthrosis
- Standing long-leg radiographs to check for any malalignment in varus and valgus
- MRI, which offers information on the chondral state and the state of the menisci but little information about the state of the graft
- CT scan with coronal, sagittal, and axial sections and 3-D reconstructions, which give the best and most precise evaluation of bone tunnel enlargement and the positions of tibial and femoral tunnel entry points on the wall of the intercondylar notch, and provide a template for planning the new tunnels.

Many authors advise the use of CT scan as a necessary tool to plan a revACLr. Marchant et al. compared plain radiography, CT scan, and MRI to evaluate bone tunnel widening, and they proved that there was superior intra- and interobserver reliability for CT scan [14]. Darren et al. proposed the REVISE ACL classification, which employs CT scan analysis to identify technical issues and guide the revision ACL treatment strategy (one or two stages) using a feasible and practical system with high internal validity, high observed agreement, and substantial inter-rater agreement [13].

Lateral X-rays are an important tool to evaluate the posterior tibial slope (PTS). An increase in PTS > 12° is associated with a higher failure rate of ACLR; for this reason, slope correction through a deflexion osteotomy (associated with ACL revision) can be considered an effective procedure to restore joint stability with a high degree of clinical satisfaction [7, 14, 15].

#### Summary

Meticulous preoperative planning is mandatory for successful revACLr. Preoperative imaging should include plain radiographs, MRI, and CT scan, and should allow the identification of the tibial and femoral tunnel positions, hardware, limb malalignment, and concomitant soft-tissue lesions, which may guide the surgeon in the decision-making process.

### If a previous ACL reconstruction with a patellar tendon fails, what’s your graft of choice? And if a previous ACL reconstruction with hamstring fails, what’s your graft of choice?

In the case of previous ACLR with a patellar tendon, 64.7% of the surgeons perform ACL revision with autologous hamstrings and 31.4% use an allograft; in the case of previous ACLR with hamstrings, 56.9% prefer to use a patellar tendon graft, 31.4% prefer an allograft, and just 11.8% opt for contralateral hamstrings.

Autografts are the most common choice overall for revACLr, including those with a patellar tendon (BPTB), quadriceps tendon–patellar bone (QTB), semitendinosus–gracilis tendons (ST-G), or an isolated multistrand semitendinosus tendon (4ST). They have greater potential healing properties and improved graft incorporation compared to the other option, albeit with the disadvantages of donor-site morbidity, variable graft sizes (especially for ST-G), anterior knee pain, and patellar fracture (for BPTB and QTB).

Allografts were selected by 31.4% in our survey, and generally included the use of BPTB, an ST-G graft, Achilles tendon bone, a quadriceps tendon patellar bone plug graft, and a tibialis anterior tendon graft. The advantages of allografts include the avoidance of donor-site morbidity associated with autograft harvesting and decreased operative time, but they present slower incorporation and potentially higher rates of failure [16].

Artificial ligaments should be carefully considered as an alternative graft option because of their poor middle- and long-term results.

The literature highlighted a higher survival rate and better clinical results for revACLr performed with autologous tendons versus an allograft, including a lower risk of re-rupture [17] and a higher satisfaction rate (Lysholm scores: 91 vs. 83, respectively) [18]. The use of allografts with a large bone block is indicated primarily if it is necessary to fill a large bone defect due to severe tunnel enlargement [2].

#### Summary

At present, there is no standard graft for ACL revision. The graft used in the original ACL reconstruction may drive the choice of the new graft. In addition, knowledge of the tunnel widening may influence the graft choice. The best results were reported with an autograft, which should be considered the graft of choice in most cases.

### What is your technique to make the femoral tunnel?

Around one-third of the surgeons (35.3%) choose the most appropriate technique on a case-by-case basis.

Just as in primary ACLR, the femoral tunnel can be made with three main techniques in ACL revision surgery: transtibial, transportal (anteromedial), and outside-in. The recent literature shows that none of these techniques is clearly superior to the others [19]. The pros and cons of the various techniques must be known by every surgeon.

Paradoxically, the creation of a new femoral tunnel is usually straightforward when the previous tunnel was malpositioned, especially when using the transportal and outside-in techniques. The greatest difficulty is encountered when the previous tunnels are correctly positioned but have become enlarged because of bone loss or fixation devices. In this situation, the outside-in technique, with its versatility, may be more useful for drilling an entirely new femoral tunnel.

#### Summary

The femoral tunnel’s position affects the tension and elongation patterns and, therefore, the working behavior of the graft. First of all, the previous tunnel must be adequately investigated; then the most appropriate technique must be chosen. It is usually tailored to the current situation, although it must be remembered that changing operating techniques is often the prerogative of experienced surgeons only. Anyway, whatever is done, the femoral tunnel must be in the most appropriate position to reduce the chance of failure.

### What is the role of lateral extra-articular tenodesis (LET) in ACL revision surgery?

Most of the surgeons (56.9%) perform a LET only in the case of a high-grade preoperative pivot-shift test.

In patients undergoing ACL revision surgery with a low degree of instability (defined as a side-to-side difference of ≤ 5 mm and a pivot-shift grade of 1 and 2 on clinical examination), a LET (modified Lemaire) is considered unnecessary because it does not influence patient-related outcomes or failure rates [20].

Conversely, various studies report reduced failure rates, improved clinical outcomes, and a reduced incidence of postoperative pivot shift in patients with a high degree of instability (defined as a side-to-side difference of over 6 mm and a pivot-shift grade of 3) in whom LET was performed in addition to ACL revision [3, 4, 20–23].

#### Summary

The extent of preoperative anterior and rotational knee laxity is an important factor to investigate in ACLR surgery. LET must be performed in patients with a high-grade pivot-shift test, especially in high-level athletes who complain of rotational instability, with the objective being to decrease the rotational laxity and increase the return to intensive sporting activity.

### What do you do in the case of a patient with primary or secondary varus (according to the Noyes classification) with pain and instability secondary to failure of a previous ACL reconstruction surgery?

In the situation described, 54.9% of the surgeons perform one-stage ACL revision surgery plus high tibial osteotomy (HTO), while 33.3% prefer staged surgery.

A high success rate of ACL revision in association with tibial opening osteotomy is reported in the literature, in particular if a concomitant reduction of the slope is performed [24, 25]. An increased tibial slope (> 12°) is regarded as a risk factor for not only a first ACL injury but also early failure after ACL reconstruction, due to the excessive tension on the graft [10].

Different osteotomy techniques are described, each with satisfactory clinical results; the most frequently performed are opening wedge osteotomy (OWHTO) and closing wedge osteotomy (CWHTO) [24].

The one-stage procedure is mainly indicated in young patients with concomitant varus alignment and medial osteoarthritis, while the staged procedure is reserved for older patients with chronic ACL deficiency [24, 26].

#### Summary

The main indication for a combination of ACL revision and HTO procedures is severe varus malalignment with medial pain associated with ACL injury and symptoms of instability.

### What meniscal injuries do you treat in ACL revision surgery?

Almost all the surgeons (98%) reported that they treat all kinds of meniscal lesions during ACL revision surgery, be they ramp, root, bucket handle, or simple lesions. Unfortunately, especially in the setting of chronic ACL lesions, meniscal injuries are often not repairable. Meniscectomy is the most frequently performed procedure according to the literature (45.1% partial meniscectomy; 30.8% meniscal repair; 5.5% meniscal allograft transplant) [27]. The failure rate for meniscus repair in the revision ACL reconstruction setting at the 2-year follow-up is quite low (< 10%), but significantly higher for medial than lateral tears [22].

#### Summary

All possible and repairable injuries must be repaired, according to the concept of “save the meniscus.”

## Conclusions

RevACLr is a complex procedure, and a thorough knowledge of all of its steps is required before performing the surgery. Many controversies still exist, and they are the focus of our study. The advice reported here represents a consensus formulated by knee-specialized Italian orthopedic surgeons with the aim of providing some key points and some practical advice to be used in daily clinical practice.

A standardized imaging protocol consisting of weight-bearing radiographs (knee and long leg), CT scan, and MRI will help with the decision-making process regarding further steps. One-stage surgery is indicated in almost all cases, with the most frequent exceptions being severe tunnel enlargement, a tunnel collision, or infection. The choice of the graft depends on the graft used previously and the tunnel enlargement. Better clinical outcomes and a lower risk of re-rupture can be expected with autografts. Some additional procedures should be performed case by case, such as lateral extra-articular tenodesis in high-grade pivot-shift knees and biplanar HTO in the case of severe coronal malalignment. The concept of “save the meniscus” is also valid for ACL revision surgery, even though a meniscal suture is often not feasible.

## Data Availability

Available from the corresponding author on reasonable request.
